# Identification and Spatial Differentiation of High-Risk Areas for Brown Bear Incidents in Yushu Prefecture, China, Using Machine Learning and Remote Sensing

**DOI:** 10.3390/ani16101489

**Published:** 2026-05-12

**Authors:** Xiaoli Guo, Jianyun Zhao, Yaxin Sun, Bo Zhai, Xinnan Ai

**Affiliations:** 1 School of Geological Engineering, Qinghai University, Xining 810016, China; 15209725207@163.com (X.G.);; 2 School of Economics and Management, Beijing Forestry University, Beijing 100083, China

**Keywords:** brown bear incidents, machine learning, high-risk area simulation, Yushu Tibetan Autonomous Prefecture

## Abstract

The Sanjiangyuan Region is among China’s most critical ecological function zones and serves as an important habitat for rare wildlife species such as brown bears and snow leopards. Driven by factors including climate change and intensified human activities, human–wildlife conflicts have become increasingly frequent on the Qinghai–Tibet Plateau, threatening the living space of both herders and wildlife. This study centers on the Yushu Tibetan Autonomous Prefecture in Qinghai Province, integrating multi-source remote sensing data with field survey data, and employs the Maximum Entropy Model (MaxEnt) MaxEnt model and the BIOMOD2 framework to simulate high-risk areas for brown bear incidents. Results indicate that the BIOMOD2 ensemble model (EMca) achieved the highest predictive accuracy, with the Random Forest (RF) model demonstrating strong robustness among individual models. Digital Elevation Model (DEM), Soil Surface Moisture (SSM), Fractional Vegetation Cover (FVC), and Human Footprint (HFP) were identified as the primary factors influencing the spatial distribution of brown bear incidents. High-risk areas exhibited significant clustering, mainly concentrated in the southern and southeastern regions of Qumalai, Nangchen, and Chindu; the eastern part of Zadoi County; and the central and southern parts of Yushu City, particularly within the elevation range of 4304–4544 m, where human activity intensity is relatively low. The core high-risk zone is located along the Tongtian River in southern Qumalai County, demonstrating strong spatial connectivity. By investigating the spatial distribution patterns and driving mechanisms of brown bear incidents in Yushu Prefecture, this study offers some references for government agencies to formulate strategies that promote harmonious coexistence between humans and nature.

## Simple Summary

In recent years, incidents involving brown bears in the Yushu Tibetan Autonomous Prefecture have occurred with increasing frequency, seriously threatening the personal safety and property of local herders. This study employs the Maximum Entropy Model (MaxEnt) and the BIOMOD2 modeling framework to simulate high-risk areas in terms of brown bear incidents. The findings demonstrate that the BIOMOD2 ensemble model provides the highest predictive accuracy. Key factors influencing brown bear incidents are Digital Elevation Model (DEM), Soil Surface Moisture (SSM), Fractional Vegetation Cover (FVC), and Human Footprint (HFP). High-risk zones are predominantly located in the central, eastern, and southeastern parts of the study area, especially in regions of moderate elevation and relatively low levels of human activity. This research provides a scientific foundation for the deployment of bear prevention measures and the development of early warning strategies, thereby supporting the harmonious coexistence of humans and nature in the Sanjiangyuan Region.

## 1. Introduction

Human–wildlife conflict is a critical global issue that constrains regional biodiversity conservation and sustainable development [1]. Brown bear incidents are particularly severe within the core coverage area of the Sanjiangyuan National Park, especially in the Yushu Tibetan Autonomous Prefecture. From 2014 to 2017, a total of 296 brown bear incidents were recorded in the Yangtze River Source Park of Sanjiangyuan National Park, with 93.6% resulting in damage to houses, 4.7% involving livestock losses, and 1.7% causing human casualties [2]. According to incomplete statistics, between May and August 2020, seven people were attacked by brown bears in Zhidoi County, Nangchen County, and Golmud City of Qinghai Province.

In recent years, wildlife-related incidents in Yushu Prefecture—including counties such as Zhidoi, Qumalai, and Zadoi—have surged dramatically, increasing from fewer than 1000 cases in 2015 to nearly 20,000 cases in 2020, representing a more than 20-fold increase [3]. This explosive growth has had a profound impact on the livelihoods and daily activities of local herders. Presently, responses to brown bear incidents predominantly rely on post-event compensation mechanisms, such as direct compensation and insurance schemes for *Pantholops hodgsonii* and *Bos grunniens*, while compensation efficiency remains low, with only about 3% of incidents covered by government compensation between 2018 and 2024 [4,5]. Due to insufficient compensation funds, difficulties in calculating conflict-related costs, high opportunity costs for submitting claims [6], and a lack of enthusiasm among herders to report cases [7], losses are not promptly made up for. These measures not only fail to promptly offset herders’ losses but are also ineffective in reducing incident occurrence at the source. Traditional studies of brown bear incidents have primarily depended on field investigations and linear statistical approaches. For instance, Xufang Han et al. [8] identified Zhidoi and Qumalai counties as the most severely affected areas in Qinghai Province through field surveys. Nonetheless, such methods are limited in capturing complex interactions among multiple factors. In recent years, machine learning techniques, with their strong capabilities in nonlinear fitting and multi-source data integration, have emerged as important tools for risk simulation. Existing studies have collected wildlife distribution data along with relevant environmental variables, applying the MaxEnt model to predict potential wildlife distribution areas [9] and to analyze the spatial distribution of *Sus scrofa* incidents [10]. By overlaying these results with human footprint data, researchers have identified high-risk zones for human–wildlife conflict, thus providing a feasible technical pathway for simulating high-risk areas for brown bear incidents in Yushu Prefecture. Additionally, some studies have used the BIOMOD2 ensemble modeling framework to predict the distribution of *Pomacea canaliculata* [11], confirming that this model can enhance the accuracy and reliability of simulations.

The Tibetan brown bear (*Ursus arctos pruinosus*), a subspecies endemic to the Qinghai–Tibet Plateau, belongs to the order Carnivora, family Ursidae, and genus Ursus. It is classified as a Class II nationally protected species in China. This subspecies is distinguished by elusive behavior, a remote habitat distribution, low population density, and primarily crepuscular and nocturnal activity patterns. Its habitat selection is closely associated with elevation and temperature [12]. Their diet exhibits seasonal variation: during the warm season, they primarily feed on *Ochotona curzoniae*, whereas in the cold season, reliance on human-stored food leads to conflict incidents [13]. Despite ongoing research efforts, studies in Yushu Prefecture continue to face limitations, including insufficient data representativeness, lack of incident records in remote pastoral areas and temporary nomadic sites, inadequate integration of species’ ecological behaviors, and neglect of spatial interactions between wildlife habitats and expanding human activities [14]. These shortcomings may introduce biases into risk simulations. Taking Yushu Prefecture as a case study, this research integrates influencing factor data—including topography, hydrology, climate, soil, vegetation, and human activities—and employs the Maximum Entropy Model (MaxEnt, v3.4.4) and BIOMOD2 (v4.2.6-2) models to simulate high-risk areas for brown bear incidents. The study further analyzes the spatial differentiation and interpretability of these high-risk areas, aiming to accurately identify the spatial distribution patterns of high-risk zones and determine the key factors influencing incident occurrence. The findings provide a scientific basis for targeted deployment of bear prevention measures—such as reinforcing herders’ houses in high-risk areas and constructing bear-proof fences—as well as for the development of regional early warning systems. These results support efforts to reduce losses to herders’ lives and property.

## 2. Materials and Methods

### 2.1. Study Area Overview and Data Sources

The Yushu Tibetan Autonomous Prefecture is situated in the heart of the Sanjiangyuan Region in Qinghai Province, China, with an average elevation exceeding 4200 m. It is bordered by the Kunlun Mountains and Bayankala Mountains to the north, the Tanggula Mountain range to the south, the alpine canyon region of western Sichuan to the east, and the northern Tibetan Plateau to the west. As the source region of the Yangtze River, Yellow River, and Lancang River, Yushu serves as the core area of Sanjiangyuan National Park, and the region is rich in flora and fauna resources. In recent years, with improvements in the ecological environment and the growth of wildlife populations, incidents involving brown bears have become increasingly frequent [15].

**Figure 1:**
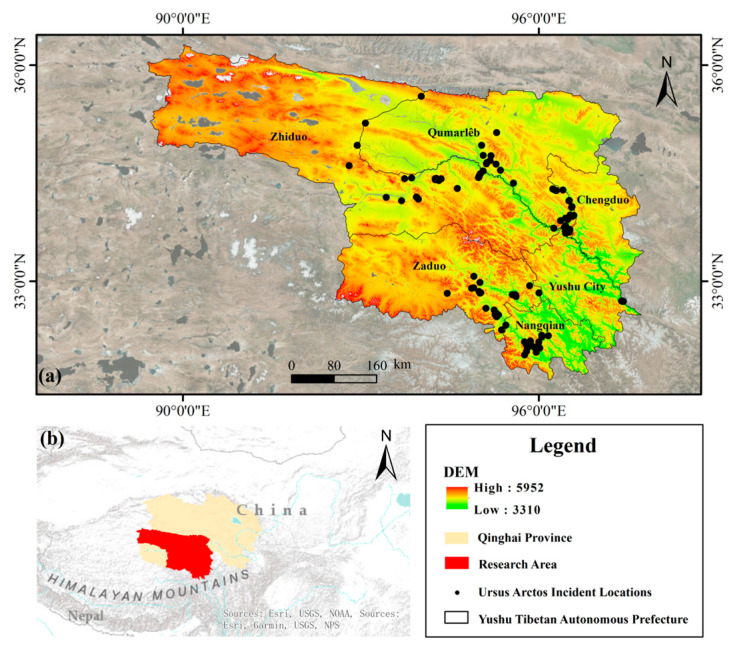
Spatial distribution of sample points in the study area. (**a**) Location map of the study area; (**b**) spatial distribution of the sample points.

Data on brown bear incident locations were collected through field investigations conducted between November 2024 and August 2025. A total of 115 incident points were identified based on multiple data sources, including herders’ oral reports, infrared camera monitoring results, and evidence of brown bear activity collected during field surveys, such as shed hair, footprints, contact marks, and feces (see Figure 1).

A total of 11 environmental and anthropogenic variables were selected as influencing factors, including Temperature (TMP), Soil Surface Moisture (SSM), Solar Radiation Intensity (SOL), Slope (SLO), Precipitation (PRE), Livestock Grazing Intensity (LHGI), LandScan Global Population Data (LandScan), Human Footprint (HFP), Fractional Vegetation Cover (FVC), Digital Elevation Model (DEM), and Aspect (ASP). Among these, data on LHGI, FVC, TMP, and SSM were obtained from the National Tibetan Plateau Data Center (https://data.tpdc.ac.cn/); SOL data were sourced from the Earth Resources Data Cloud platform (www.gis5g.com); PRE data were derived from the ERA5-Land dataset [16]; population data were obtained from the LandScan Population Data Explorer (https://landscan.ornl.gov/); and the HFP dataset was developed by the UEMM team [17]. DEM data were acquired from the Shuttle Radar Topography Mission (SRTM) 90 m DEM provided by the United States Geological Survey [18], from which SLO and ASP were further derived. To ensure spatial consistency, all variables were resampled to a spatial resolution of 1 km, and the coordinate system was unified to EPSG: 4326.

### 2.2. Modeling Procedure

This study employs the MaxEnt model and the BIOMOD2 ensemble modeling framework to predict the potential distribution of brown bear incidents, simulating high-risk areas within the study region. Based on the principle of maximum entropy, MaxEnt is a density estimation and species distribution modeling approach that predicts potential geographic distributions by integrating known occurrence data with environmental constraints [19]. In this research, it is used to simulate the potential distribution of brown bear incidents [20] and to quantify the contribution of each influencing factor, where higher contribution values indicate greater importance in determining the distribution patterns [21]. Model performance is evaluated using the Receiver Operating Characteristic (ROC) curve and the Area Under the Curve (AUC) [22]. The BIOMOD2 platform, developed in R, facilitates species distribution modeling by integrating multiple algorithms and input types, including endangered species, invasive species, and habitat data. It incorporates a diverse array of modeling approaches, including Generalized Linear Models (GLM), Artificial Neural Networks (ANN), Generalized Additive Models (GAM), Multivariate Adaptive Regression Splines (MARS), Classification Tree Analysis (CTA), Flexible Discriminant Analysis (FDA), Surface Range Envelope (SRE), Generalized Boosted Regression Models (GBM), Random Forest (RF), and MaxEnt [23]. By repeatedly partitioning independent samples and evaluating model outputs, BIOMOD2 selects optimal models and mitigates issues such as data redundancy caused by multiple variables. There are two types of ensemble methods for single models, Ensemble Model-CA (EMca) and Ensemble Model-Weighted Mean (EMmean) [24]. EMca integrates the predictions of different models into a final classification result, typically determining the category of each pixel through a voting mechanism. EMmean calculates the weighted average of predictions from all models, with weights assigned based on the performance of each model [25]. Model accuracy is assessed using Kappa, AUC, True Skill Statistics (TSS), and Critical Success Index (CSI) [26]. In addition, the Jackknife method is applied to evaluate the relative importance of environmental variables.

To investigate the spatial heterogeneity of brown bear incidents, this study applies the K-means clustering method [27], spatial autocorrelation analysis [28], and spatial structure analysis. Furthermore, the Extreme Gradient Boosting (XGBoost) model [29] combined with Shapley Additive Explanations (SHAP) [30] is used to interpret the contributions of influential factors and to explore the interactions between human activities and key environmental variables. Local Moran’s I is used to identify clustering or dispersion patterns at the local scale, while Global Moran’s I is used to measure overall spatial autocorrelation. LISA, a general class of local spatial association indicators, can decompose global spatial autocorrelation metrics such as Moran’s I into the contributions of individual observations. This enables the detection of local spatial hotspots and nonstationarity, as well as the assessment of each spatial unit’s impact on global statistical results and the identification of spatial outliers [31].

In the spatial structure analysis, the Shape Index is used to quantify morphological complexity; higher values indicate more complex boundaries and greater fragmentation. For connectivity analysis, the Nearest Neighbor Index (NNI) is employed to determine spatial distribution patterns: NNI < 1 indicates clustering, NNI > 1 indicates dispersion, and NNI = 1 indicates a random distribution [32].

## 3. Results

### 3.1. Analysis of Model Simulation Results

Based on the variables and incident location data obtained from field investigations, both the MaxEnt and BIOMOD2 models were constructed to simulate the potential distribution of conflict incidents. The results showed that the MaxEnt model achieved an average test AUC of 0.961 for the ROC curve, and the model results were stable across multiple iterations, with the omission rate curve closely following the ideal prediction curve, indicating a good fit without obvious overfitting or underfitting. The results of the Jackknife test [33] revealed substantial differences in the contributions of individual variables. Notably, elevation and human footprint showed higher gains, indicating that they are the dominant factors influencing the spatial distribution of brown bear incidents (see Supplementary Materials). The variable contribution rates and importance indices of the model are shown in Table 1. Elevation, solar radiation intensity, and human footprint together accounted for 72.4% of the total contribution, with elevation ranking highest in both percent contribution and permutation importance. The human footprint variable reflects dispersed human activities such as grazing and “summer camps” (temporary residences), which are typically in areas with low population density, whereas the population variable mainly reflects population aggregation. This also explains why the permutation importance of the population data was only 0.3%.

**Figure 2:**
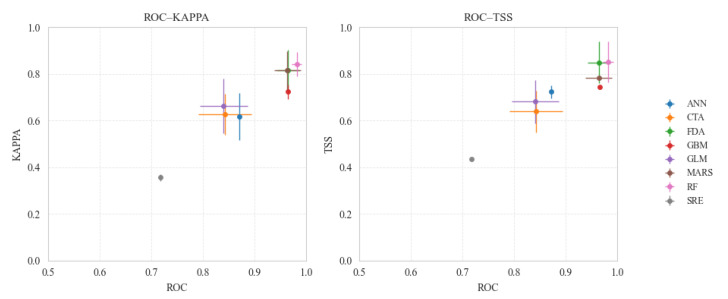
ROC-KAPPA evaluation chart and ROC-TSS indicator chart.

**Figure 3:**
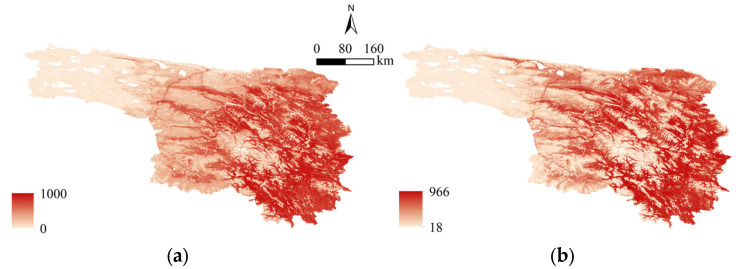
Simulation of potential distribution of bear-caused incidents based on optimal individual and ensemble models. (**a**) RF; (**b**) EMca.

The BIOMOD2 platform implemented in R was utilized to run a suite of individual models [34,35]. Among the various models, as depicted in Figure 2, Kappa values across these models ranged from 0.43 to 0.99, TSS values ranged from 0.42 to 0.99. Notably, the RF model achieved the best performance among all single models (Table 2). The RF model also demonstrated strong robustness in the complex high-altitude terrain and diverse ecological gradients characteristic of Yushu Prefecture. When integrating the above eight individual models, both the EMmean and EMca ensemble algorithms performed excellently, with their respective metrics shown in Table 3. The simulation results of brown bear incidents in Yushu Prefecture using the independent RF model and the EMca ensemble model are shown in Figure 3. The spatial distributions predicted by the two models were highly consistent, and the incidents exhibited pronounced spatial heterogeneity, with high-risk areas concentrated in the central, eastern, and southeastern parts of Yushu Prefecture, showing dense distributions, while the northwest and western areas were more sparsely distributed. Overall, there is a decreasing trend from the southeast to the northwest. In summary, the ensemble models outperformed the individual models, and thus, subsequent spatial analyses were based on the optimal ensemble results.

### 3.2. Spatial Distribution Characteristics of Brown Bear Incidents

#### 3.2.1. Cluster Analysis

**Figure 4:**
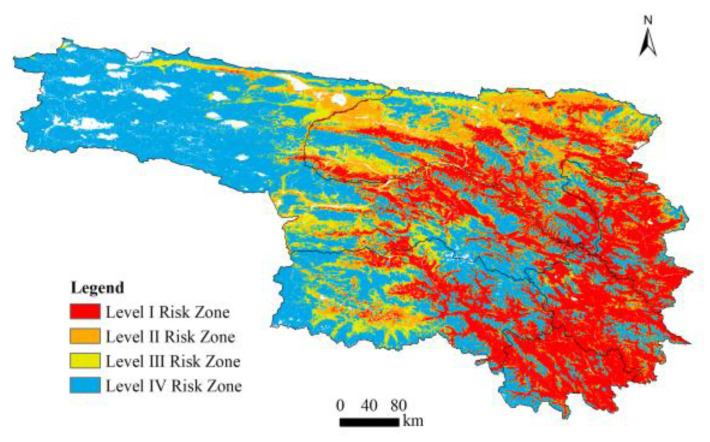
K-means clustering analysis.

The K-means clustering method was applied to the ensemble model results (Figure 4), classifying the study area into four risk levels: Level I (highest risk), Level II, Level III, and Level IV (lowest risk). Level I risk areas are predominantly distributed in the southern and southeastern parts of Qumalai, Nangchen, Chindu, eastern Zadoi County, and the central and southern parts of Yushu City, primarily within elevations of 4304–4544 m. Level II risk areas are mainly situated in eastern Zhidoi County, northern and southwestern Qumalai County, and western Zadoi County. These areas share similar natural conditions with Level I regions but are characterized by more seasonal and dispersed grazing activities, leading to a moderate risk of conflict. Level III risk areas are scattered and more strongly influenced by random factors, while Level IV areas are primarily located in the western parts of the Qumalai, Zhidoi, and Zadoi counties. These regions are characterized by high elevation or limited accessibility, with minimal human activity and scarce food sources, resulting in the lowest conflict risk.

#### 3.2.2. Spatial Autocorrelation Analysis

**Figure 5:**
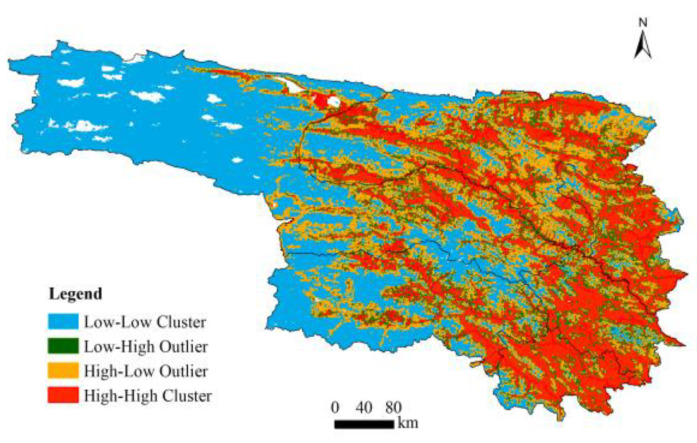
Local autocorrelation analysis.

Global Moran’s I was utilized to assess overall spatial autocorrelation based on the BIOMOD2 ensemble results, while LISA was employed to identify local clustering patterns (Figure 5). The study area was classified into four cluster types: High–High (HH), Low–Low (LL), Low–High (LH), and High–Low (HL). The Global Moran’s I value of 0.953 indicates strong positive spatial autocorrelation, suggesting that brown bear conflict risk is highly clustered rather than randomly distributed, and is influenced by continuous environmental gradients or spatial processes. According to the results of local spatial autocorrelation, HH and LL clusters account for 44.3% and 51.8% of the total area, respectively, while LH and HL outlier categories comprise only 3.9% in total. This indicates that the spatial risk pattern is stable and there are no large areas of isolated values. The spatial structure of brown bear incident risk in Yushu Prefecture is characterized by higher risk in the east, lower risk in the west, and a contiguous distribution.

#### 3.2.3. Spatial Structure of High-Risk Areas

**Figure 6:**
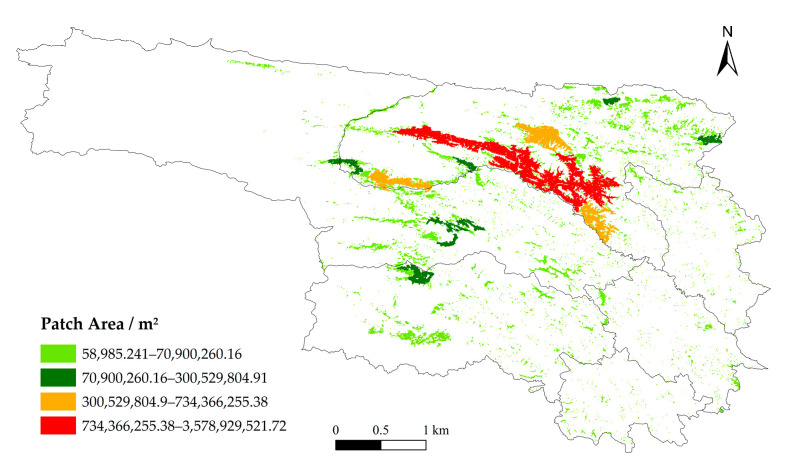
Patch area.

**Figure 7:**
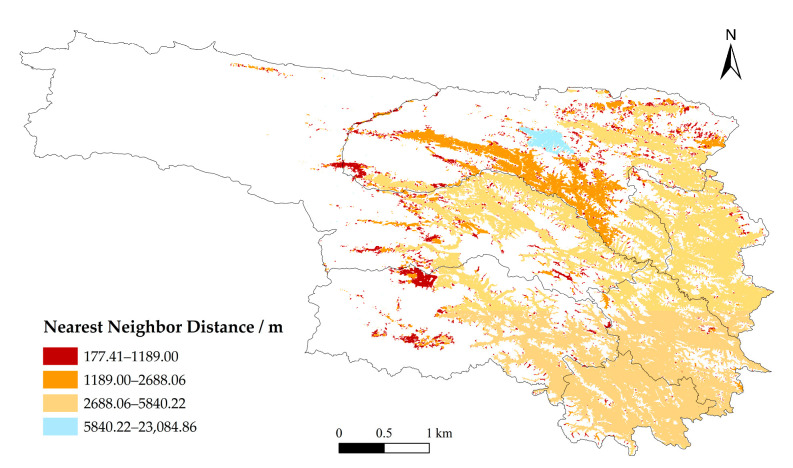
Nearest neighbor distance.

**Figure 8:**
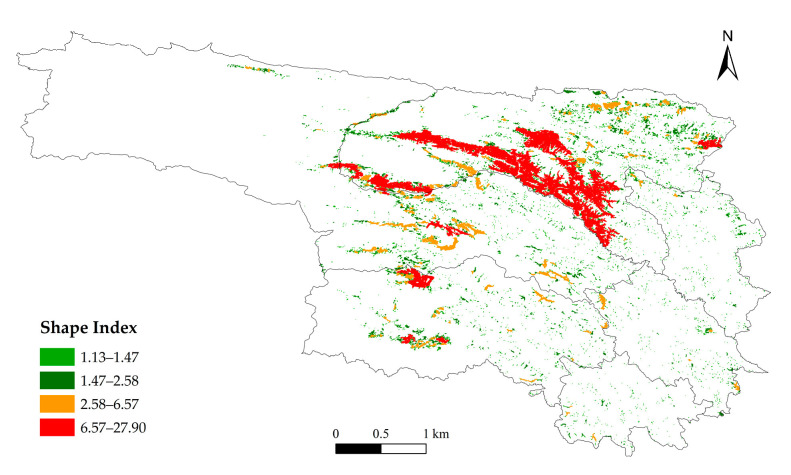
Shape index.

Analysis of Level I risk patches reveals a heterogeneous clustering pattern. Numerous small patches are widely distributed in peripheral areas, while a few large patches are concentrated in the southern and western parts of Qumalai County, particularly along the Tongtian River (Figure 6). In these belt-shaped regions, patch spacing is minimal, and clustering is pronounced, whereas in peripheral areas, patch spacing increases and connectivity decreases (Figure 7). Patch boundaries in southern and southwestern Qumalai, northern Zhidoi, and northwestern Zadoi are complex and irregular, suggesting heightened edge effects and landscape fragmentation due to terrain constraints or human activities. In contrast, peripheral patches are generally more regular in shape but spatially dispersed, making it difficult to form continuous structures (Figure 8). Overall, the Tongtian River corridor in southern Qumalai County represents the core high-risk area for brown bear incidents and exhibits potential for further spatial expansion.

### 3.3. Driving Mechanisms and Interpretability Analysis

#### 3.3.1. Relative Importance and Response of Variables

Multicollinearity among variables was assessed using stepwise regression, with variables exhibiting Variance Inflation Factors (VIF) [36] greater than 10 excluded from further analysis [37]. As shown in Table 4, all variables passed the test and were retained. Five common models were compared (Table 5), and the Extreme Gradient Boosting (XGBoost) model demonstrated the best performance, exhibiting lower mean squared error (MSE) and higher R^2^ values. These results indicate that XGBoost is particularly well-suited for analyzing the influence of variables on brown bear incident risk.

**Figure 9:**
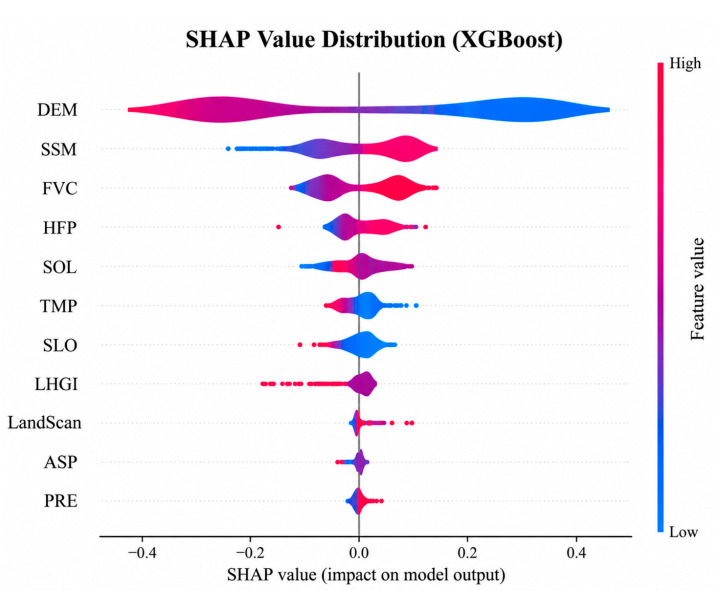
SHAP value distribution.

SHAP can quantify the contribution of feature variables to model prediction results under the distribution of observed data. Figure 9 presents a summary plot of SHAP values, with variables ranked by importance from top to bottom. Among them, elevation exhibits the widest range of distribution, indicating that its impact on brown bear incidents varies greatly across different regions and displays significant spatial heterogeneity. Soil moisture and vegetation cover also show considerable ranges of fluctuation. The human footprint variable demonstrates a pronounced bipolar effect on brown bear incidents in certain ranges, suggesting that human activity intensity can either promote the occurrence of incidents or, to some extent, inhibit brown bear activities. In addition, other factors have overall narrower distributions, indicating that their influence is relatively minor across most areas. Therefore, terrain conditions, ecological environment factors, and the intensity of human activities are the key drivers of the spatial variation in brown bear incident risk. The distribution of SHAP values for different variables exhibits notable differences, reflecting the complex nonlinear responses and pronounced spatial heterogeneity of brown bear incident risk to environmental factors.

**Figure 10:**
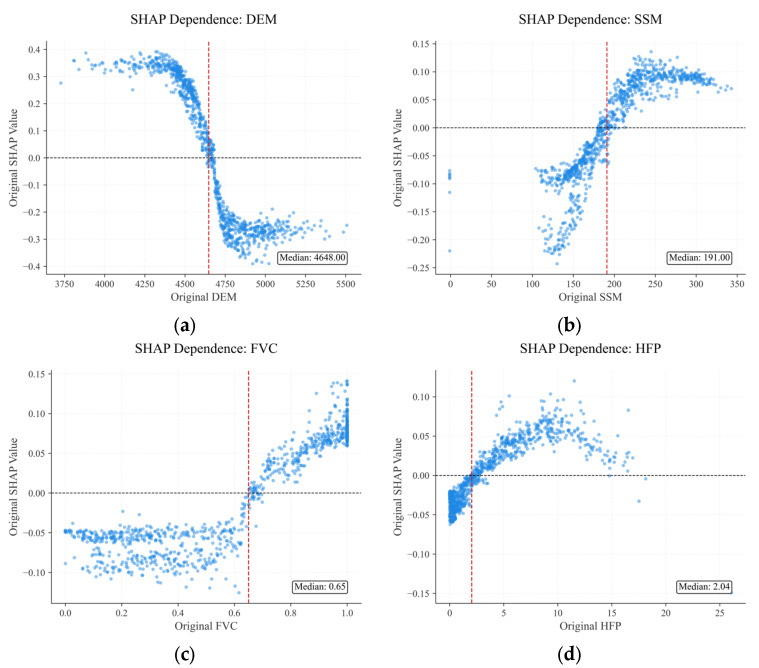
SHAP dependence plots for key variables. (**a**) DEM; (**b**) SSM; (**c**) FVC; (**d**) HFP.

Building on the assessment of variable importance, Figure 10 presents a detailed analysis of the relationships between key variables and the risk of brown bear incidents. DEM demonstrates a distinct nonlinear association with incident risk. In relatively low-elevation areas, increasing elevation promotes the risk of brown bear incidents; however, as elevation continues to rise, SHAP values turn negative, indicating that high-elevation areas exert a suppressive effect on incident risk. Thus, within the study area, brown bear incident risk peaks at moderate elevations and declines significantly at extreme elevations. SSM exerts a suppressive effect on incident risk at low levels, but as SSM increases, the risk rises markedly, indicating that wetter environments are associated with higher probabilities of brown bear incidents. FVC has a relatively minor influence at low to moderate levels, but its promoting effect on incident risk becomes more substantial as FVC increases to higher levels. HFP contributes most to risk at moderate levels but decreases in areas with high-intensity human activity, indicating that low to moderate human activity may increase the likelihood of incidents, whereas excessive disturbance inhibits brown bear occurrences.

#### 3.3.2. Response Relationships of Key Variables

**Figure 11:**
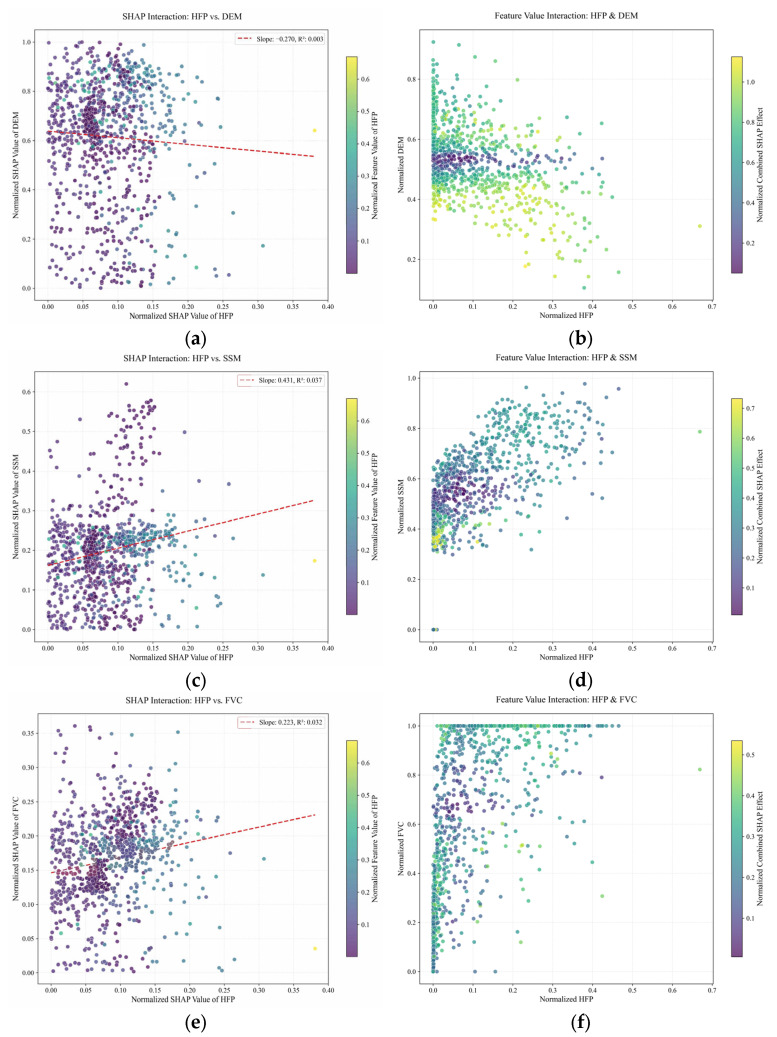
Interactive effects between anthropogenic and natural factors. (**a**,**b**) HFP and DEM; (**c**,**d**) HFP and SSM; (**e**,**f**) HFP and FVC.

Given that HFP directly reflects the intensity of human activity, this study selects three dominant environmental variables—DEM, SSM, and FVC—to investigate their interactions with HFP in influencing brown bear incidents (Figure 11). The interaction between HFP and DEM exhibits a weak negative correlation in terms of contribution. At high elevations, even in the presence of HFP, the contribution to incident risk is significantly suppressed, whereas at moderate elevations, human activities are more likely to trigger brown bear incidents (Figure 11a). In terms of feature value combinations, the strongest coupling occurs where HFP is low and elevation is moderate (Figure 11b). At the SHAP value level, as HFP increases to 0.4, the SHAP value of SSM rises to 0.2, confirming that SSM exerts a positive amplification effect on incident risk when human activity is involved (Figure 11c). In the feature space, the lightest-colored areas correspond to low HFP and moderate SSM; however, due to the very low intensity of human activity in these areas, the absolute number of incidents remains small, and thus their overall contribution to risk is not fully realized (Figure 11d). The SHAP contribution of FVC increases with rising HFP, indicating that vegetation—serving as both a resource and shelter—continuously elevates the risk of brown bear incidents under increasing human activity (Figure 11e). The strongest interaction between FVC and HFP occurs in areas with moderate FVC and low HFP, where suitable habitat conditions overlap spatially with human activities (Figure 11f). Overall, brown bear incidents are not driven by a single factor but are the result of the combined effects of DEM, SSM, and FVC under the influence of human activity.

## 4. Discussion

This study draws on field investigations conducted in the Yushu Tibetan Autonomous Prefecture, from which 115 spatial records of brown bear incidents were collected. By combining 11 variables with multiple modeling approaches, high-risk areas for brown bear incidents were effectively simulated. The BIOMOD2 ensemble model integrates the strengths of individual models and mitigates potential biases inherent in single-model approaches; this is also consistent with most studies [38]. The eastern region of Yushu Prefecture is characterized by low undulating mountains, while the western region is distinguished by higher elevations and more complex terrain [39]. Risk zoning results reveal a distinct spatial pattern of brown bear incident risk, characterized as “higher in the east and lower in the west,” with high-risk areas predominantly located in the central, eastern, and southeastern parts of the study region. Level I and II risk areas are predominantly distributed across mid-to-high elevation zones with frequent pastoral activities. In these areas, seasonal transhumance practices (winter–summer grazing rotation) result in heightened human activity intensity during specific periods. Defensive attacks are more common in summer, while in winter, incidents often involve bears entering houses. These high-risk areas are primarily within elevations of 4304–4544 m. This finding aligns with previous research indicating that brown bear climate refugia in the Sanjiangyuan Region are mainly located between 4300 and 4600 m [40]. These regions are characterized by low human footprint values and limited urbanization, serving as key grazing zones where human activities spatially overlap with natural bear habitats, thus forming core conflict areas. Level III and IV risk areas are primarily situated in remote plateau hinterlands far from settlements, where incidents are more likely to occur randomly. The gradual spatial transition from high to low-risk areas reflects a continuous variation in brown bear incident risk, underscoring the rationality and practical significance of the risk classification. Nevertheless, due to the inherent randomness of such incidents, local inconsistencies with model predictions may arise. Therefore, the results of this study should be considered as a macro-scale risk identification tool rather than a precise predictor at the local level.

From the perspective of spatial distribution characteristics, risk patches display a hierarchical pattern dominated by numerous small patches and a few large patches. Large high-risk patches are primarily concentrated in the central–northern part of Yushu, forming a distinct belt-like aggregation near the boundary between Qumalai County and Zhidoi County, especially along the Tongtian River. These belt-shaped areas are characterized by short distances between patches, high connectivity, and strong potential for spatial expansion, making them core zones of risk evolution. After hibernation, brown bears experience high energy consumption, and natural food sources are often insufficient to meet their survival needs. As a result, their foraging range expands, increasing the likelihood of direct encounters with humans and resulting in conflict incidents [41]. This finding aligns with the results of this study, which indicate that the core area possesses significant expansion potential. Patch boundaries within these areas are complex and irregular, indicating that risk expansion is driven by multiple factors and exhibits dynamic spatial behavior. In contrast, peripheral patches are more dispersed, with larger distances that hinder the formation of continuous structures.

High-risk areas are predominantly distributed in ecological zones with moderate elevation, high SSM, moderate FVC, and low HFP. Moderate elevation and higher soil moisture are often associated with environments rich in water and vegetation resources. Previous studies have pointed out that herders typically choose to pitch their tents in places abundant in water and grass, which are ideal for grazing and daily life [42]. These locations not only provide better food sources and shelter for brown bears but also coincide with grazing areas for herders, resulting in a higher risk of human–bear encounters, which is consistent with the conclusions of this study. In regions with low human activity intensity, brown bears are more likely to access high-energy food sources stored in residences, such as flour, yak butter, meat, and livestock feed. These resources are typically stored in herders’ winter houses (“winter camps”), which are frequently unoccupied during summer grazing periods, making them prime targets for bear intrusion [43]. In contrast, in areas with high-intensity human activity, strong anthropogenic disturbance may suppress brown bear activities. This aligns with related research indicating that brown bears clearly avoid areas of intense human activity, with such disturbances compressing their activity range and reducing the frequency of close-range encounters [44]. In recent years, with the implementation of settlement policies, the construction of poverty alleviation housing, and the contracting of grasslands to individual households, most herders have adopted a rotational grazing pattern—settling in fixed residences during winter and living in tents while grazing in summer. These “winter houses” serve as permanent residences for storing large quantities of food and daily necessities, but remain vacant for extended periods during the summer, making them prime targets for brown bears seeking food indoors. This seasonal mismatch in spatial use is a key factor intensifying brown bear incidents, which is consistent with the findings of Zhang Qiangyuan et al. [45]. It also supports Li et al.’s conclusion that the intensity of brown bear intrusions into residences is significantly negatively correlated with human activity intensity on a daily timescale [46].

Fundamentally, brown bear incidents reflect spatial conflicts and resource competition between ecological conservation and human development. The most effective way to mitigate such conflicts is through proactive prevention measures and the establishment of comprehensive legal and regulatory frameworks to safeguard local livelihoods and promote human–bear coexistence [47] and keep brown bear incidents within a tolerable range [48]. First, proactive installation of protective fencing should be implemented to reduce direct conflicts [46]. Second, management and monitoring of herders’ residences during seasonal migration periods should be reinforced, alongside scientific guidance on grazing practices and the appropriate defensive measures. Public awareness and education on bear prevention should also be enhanced. Additionally, governments should establish efficient, standardized, and transparent compensation mechanisms for bear-related damages, with simplified claim procedures to improve compensation effectiveness [7].

It should be noted that field investigations in this study were primarily conducted in Zhidoi and Qumalai counties, resulting in relatively limited sample data from other counties and a spatial imbalance in the training dataset. The results indicate that core high-risk areas are mainly concentrated along the Tongtian River in southern Qumalai County, and the findings are more applicable to key monitored regions, while results for other areas require further validation. Regarding human activity data, the HFP index used in this study mainly reflects macro-scale patterns. However, herders’ activities exhibit strong seasonal variability—for example, during the caterpillar fungus harvesting season and summer grazing periods—potentially increasing human–bear encounters and influencing the spatiotemporal distribution of incident risk. As a result, the model may underestimate certain seasonally high-risk areas. Additionally, this study focuses primarily on environmental factors and human activity intensity, without incorporating socio-ecological variables such as bear population dynamics, food resource fluctuations, and herders’ self-protection measures, which may introduce uncertainty into model predictions. Future research should expand the survey scope, incorporate seasonal variations, and integrate socio-ecological and behavioral data to further improve model accuracy and applicability.

## 5. Conclusions

This study focuses on the Yushu Tibetan Autonomous Prefecture, integrating multi-source data with field survey samples and employing a suite of machine learning models to simulate high-risk areas for brown bear incidents. The research also investigates the influence of various factors on brown bear incidents, as well as the interactions between human activity and environmental variables. The results indicate that high-risk areas for brown bear incidents are predominantly distributed in the southern and southeastern parts of Qumalai, Nangchen, and Chindu; the eastern part of Zadoi County; and the central and southern parts of Yushu City, particularly within mid-elevation zones. These high-risk areas exhibit a spatial pattern characterized by higher risk in the east and lower risk in the west, with a contiguous distribution. The core high-risk zone is located along the Tongtian River in southern Qumalai County, featuring strong connectivity, while peripheral areas show a high degree of fragmentation. The spatial pattern of brown bear incident risk in Yushu Prefecture is shaped by the interplay between natural plateau habitat conditions and dispersed pastoral activities. DEM, SSM, FVC, and HFP are identified as the primary factors influencing the occurrence of brown bear incidents.

## Figures and Tables

**Table 1:** Contribution rate and permutation importance of variables to the MaxEnt model.

Variable	Percent Contribution (%)	Permutation Importance (%)
DEM	32.7	38.9
SOL	20.6	20.8
HFP	19.1	9.8
FVC	7.6	10.1
ASP	4.7	3.6
TMP	4.4	4.9
SSM	2.9	1.5
LandScan	2.6	0.3
LHGI	2.5	3.7
SLO	1.5	2
PRE	1.5	4.3

**Table 2:** Accuracy of individual models.

Model	ROC	TSS	Kappa	CSI
RF	0.999	0.982	0.974	0.982
GLM	0.989	0.938	0.911	0.938
ANN	0.993	0.947	0.924	0.947
CTA	0.978	0.868	0.845	0.898
FDA	0.984	0.922	0.922	0.948
GBM	0.999	0.973	0.961	0.974
MARS	0.997	0.973	0.961	0.973
SRE	0.855	0.709	0.644	0.741

**Table 3:** Prediction accuracy of the ensemble model.

Model	Kappa	TSS	AUC	CSI
EMmean	0.936	0.947	0.996	0.957
EMca	0.936	0.956	0.998	0.958

**Table 4:** Multicollinearity analysis.

Variable	VIF
DEM	5.2534
SOL	3.8806
HFP	5.4310
FVC	4.1288
ASP	1.0440
TMP	1.7927
SSM	5.2561
LandScan	2.1568
LHGI	1.9637
SLO	1.3777
PRE	4.7592

**Table 5:** Prediction accuracy of each model.

Model	RF	XGBoost	LightGBM	LinearRegression	SVR
MSE	0.0057	0.0029	0.0029	0.0289	0.0056
R^2^	0.9548	0.9768	0.9767	0.7717	0.9559

## Data Availability

The original contributions presented in this study are included in the article/Supplementary Materials. Further inquiries can be directed to the corresponding author.

## Supplementary Materials

The following supporting information can be downloaded at: https://www.mdpi.com/article/10.3390/ani16101489/s1, Figure S1: Jackknife analysis of variables; Figure S2: Validation of MaxEnt model accuracy.
