# Supplementary figures and images for: Identification and Spatial Differentiation of High-Risk Areas for Brown Bear Incidents in Yushu Prefecture, China, Using Machine Learning and Remote Sensing
Source: Animals (Basel). 2026 May 12;16(10):1489. doi: 10.3390/ani16101489 (PMC13203201; doi:10.3390/ani16101489)

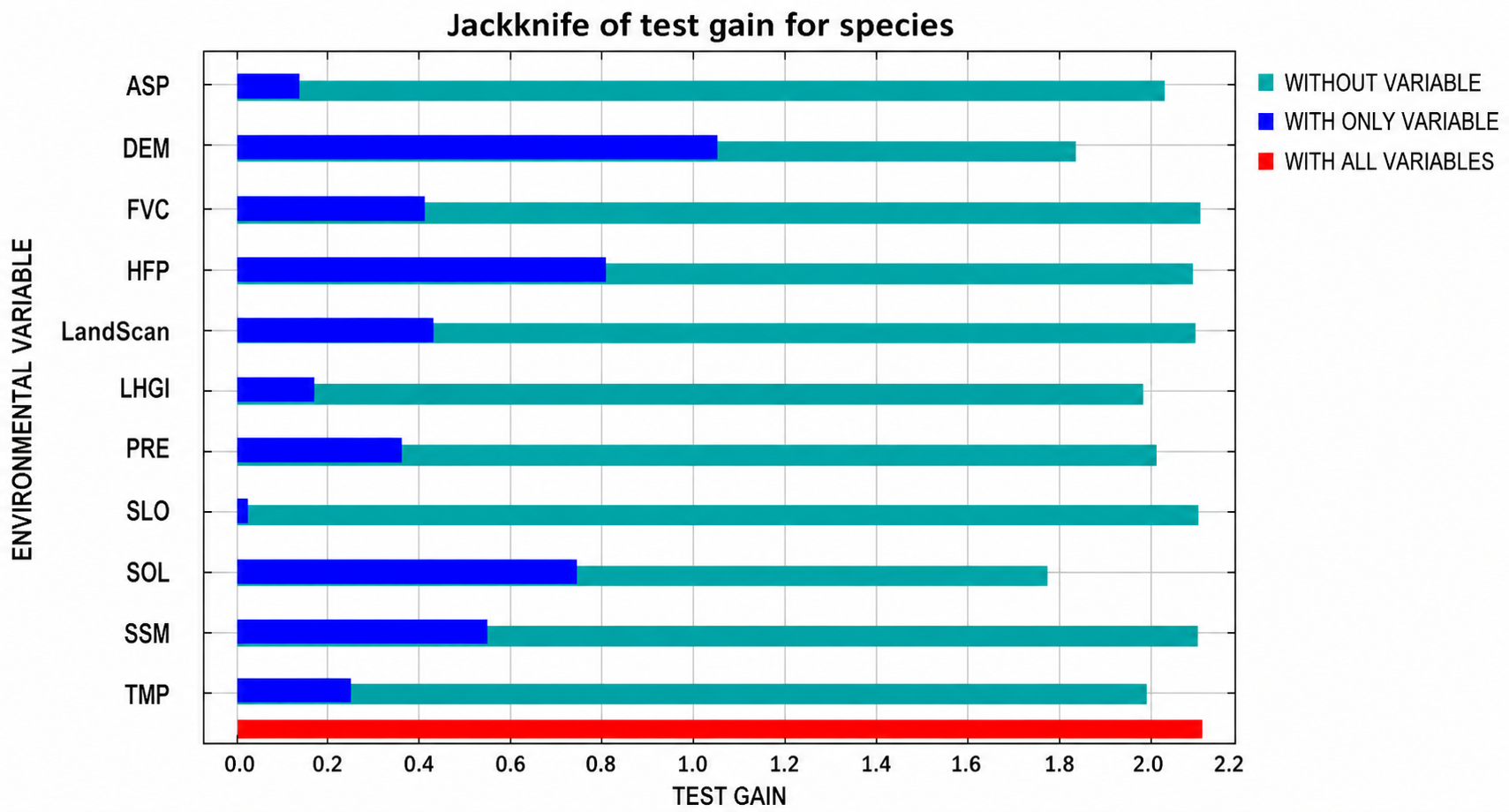

Supplement: Supplementary file 1 [file animals-16-01489-s001.zip › Supplementary Files/Jackknife analysis of variables.png]

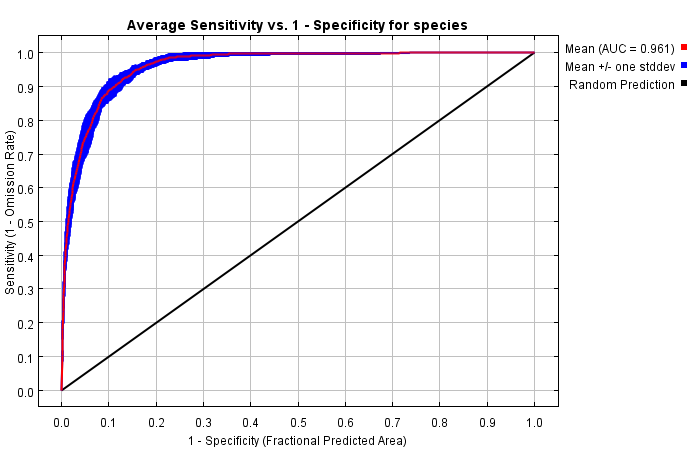

Supplement: Supplementary file 1 [file animals-16-01489-s001.zip › Supplementary Files/Validation of MaxEnt model accuracy.png]
